# Lithium-Induced Reorientation of Few-Layer MoS_2_ Films

**DOI:** 10.1021/acs.chemmater.3c00669

**Published:** 2023-08-02

**Authors:** Michaela Sojková, Igor Píš, Jana Hrdá, Tatiana Vojteková, Lenka Pribusová Slušná, Karol Vegso, Peter Siffalovic, Peter Nadazdy, Edmund Dobročka, Miloš Krbal, Paul J. Fons, Frans Munnik, Elena Magnano, Martin Hulman, Federica Bondino

**Affiliations:** † Institute of Electrical Engineering, SAS, Dúbravská cesta 9, 841 04 Bratislava, Slovakia; ‡ IOM-CNR, Istituto Officina dei Materiali, S.S. 14 km − 163.5, Basovizza, Trieste 34149, Italy; § Institute of Physics, Slovak Academy of Sciences, Dúbravská cesta 9, 84511 Bratislava, Slovakia; ∥ Centre for Advanced Materials Application (CEMEA), Slovak Academy of Sciences, Dúbravská cesta 5807/9, 84511 Bratislava, Slovakia; ⊥ Center of Materials and Nanotechnologies (CEMNAT), Faculty of Chemical Technology, University of Pardubice, Legions Square 565, 530 02 Pardubice, Czech Republic; # Department of Electronics and Electrical Engineering, Faculty of Science and Technology, Keio University, 223-8522 3-14-1 Hiyoshi, Kohoku-ku, Yokohama, Kanagawa 223-8522, Japan; ∇ Device Technology Research Institute, National Institute of Advanced Industrial Science and Technology, 1-1-1 Umezono, Tsukuba, 305-8568 Ibaraki, Japan; ○ Helmholtz-Zentrum Dresden-Rossendorf, e.V. Bautzner Landstrasse 400, D-01328 Dresden, Germany; ◆ Department of Physics, University of Johannesburg, Auckland Park, PO Box 524, 2006 Johannesburg, South Africa

## Abstract

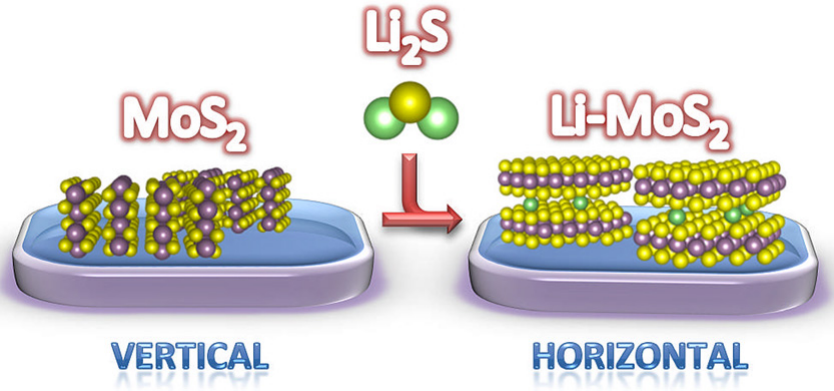

Molybdenum disulfide (MoS_2_) few-layer films have gained considerable attention for their possible applications in electronics and optics and also as a promising material for energy conversion and storage. Intercalating alkali metals, such as lithium, offers the opportunity to engineer the electronic properties of MoS_2_. However, the influence of lithium on the growth of MoS_2_ layers has not been fully explored. Here, we have studied how lithium affects the structural and optical properties of the MoS_2_ few-layer films prepared using a new method based on one-zone sulfurization with Li_2_S as a source of lithium. This method enables incorporation of Li into octahedral and tetrahedral sites of the already prepared MoS_2_ films or during MoS_2_ formation. Our results discover an important effect of lithium promoting the epitaxial growth and horizontal alignment of the films. Moreover, we have observed a vertical-to-horizontal reorientation in vertically aligned MoS_2_ films upon lithiation. The measurements show long-term stability and preserved chemical composition of the horizontally aligned Li-doped MoS_2_.

## Introduction

Molybdenum disulfide (MoS_2_) has emerged as a highly promising material for a variety applications, including transparent electronics,^1^ nanotribology,^2^ lithium-ion batteries,^3,4^ and catalysis.^5−7^ MoS_2_ is a layered transition metal dichalcogenide changing from an indirect to a direct semiconductor as film thickness approaches a monolayer.^8^ MoS_2_ is analogous in structure to graphite, where Mo and S atoms are arranged in a sandwich structure by the S–Mo–S sequence of covalent bonds^9^ with interlayer interactions being governed by relatively weak van der Waals forces. A variety of strategies have been applied to tune the physical, chemical, and electronic properties of MoS_2_. These include dimensional sizing (reducing to atomic-layer thickness as well as nanoscale reduction of lateral size), tuning the stacking order, atomic structure, type and density of structural defects, and ion intercalation.^10^ In general, MoS_2_ films can have two different crystallographic orientations: the basal planes can be oriented parallel (horizontal alignment) or perpendicular (vertical alignment) to the substrate. The presence of basal planes or edge sites is important for anticipated applications of this material. Horizontally aligned films are suitable for electronics^11,12^ and optoelectronics,^13^ such as ultrafast tunnel diodes^14^ or photodetectors^15^ for use in harsh environments. Vertically aligned MoS_2_ is a promising candidate for substituting noble-metal catalysts in electrochemical hydrogen production^16^ conversion of CO_2_ to energy-rich products,^17^ water disinfection,^18^ water splitting,^19^ or solar cells.^20^ The orientation should also be considered when MoS_2_ is interfaced with other materials, such as stacked 2D material heterostructures or electric contacts in transistors.

Several methods can be used to fabricate the MoS_2_ monolayer or few-layers films. The most commonly used methods are bulk crystals exfoliation^21^ and chemical vapor deposition.^22^ However, the production of high-quality large-area films is still a challenge. One of the suitable methods is sulfurization of pre-deposited molybdenum or molybdenum oxide layers. Moreover, this method allows the fabrication of MoS_2_ films with both orientations. The growth of vertical MoS_2_ is often connected to rapid sulfurization in a two-zone furnace.^23−25^ Several parameters control the final layer orientation in the sulfurized layers. According to Kong et al.,^24^ the reaction requires sulfur diffusion into the Mo film to convert it into sulfide. The mass transport along the layers through van der Waals gaps is much faster than across the layers in MoS_2_. Consequently, the layers tend to be perpendicular to the substrate. Jung et al.^23^ demonstrated that the thickness of the initial Mo layer is a critical parameter that determines the growth directions. Vertical growth dominates with thicker (≳3 nm) Mo layers, while the horizontal growth occurs in thinner layers. Sojkova et al.^26,27^ employed the one-zone sulfurization method for the growth of MoS_2_ films. In this simplified sulfurization technique, the Mo substrate and the sulfur powder are placed in close proximity to each other at the center of a one-zone furnace. They are exposed to the same temperature without additional control over the sulfur temperature. Using this method and high annealing rate (25 °C/min), it was noted that the thickness of the pre-deposited Mo layer is the determining factor for film orientation regardless of the substrate used, annealing temperature, or duration. Another factor that influences the layer orientation is the sulfur evaporation rate. Higher sulfur vapor pressure during sulfurization leads to the formation of vertical MoS_2_ layers, while slower sulfur evaporation results in horizontally aligned layers even in the case of a thicker initial Mo layer. This finding was confirmed by an in situ X-ray scattering study.^28^

One of the unique properties of MoS_2_ (and similar layered materials) is the ability to intercalate guest species into their van der Waals gaps. Intercalation may change the electronic structure and optical and electrical properties directly by electron doping or indirectly by inducing a phase transition or structural and compositional disorder.^29^ For the case of lithium (Li) intercalation, the MoS_2_ structure allows a fast diffusion path for the movement of Li ions in the absence of a significant volume change.^30^ Li intercalation may lead to exfoliation but also to conversion of the semiconducting 2H phase to the metallic 1T′ phase.^21^ Interestingly, recent studies have shown enhanced MoS_2_ catalytic performance following Li intercalation, particularly, in the hydrogen evolution reactions^31^ and CO_2_ reduction.^17,32^

Lithium is typically incorporated into the MoS_2_ layer electrochemically^33^ or using lithium reactants, such as the widely adopted *n*-butyllithium diluted in hexane^34^ or lithium naphtalenide.^35,36^ Wet chemical methods are mostly applied for exfoliation of bulk material to produce mono-layers or few-layer flakes. MoS_2_ can also be intercalated in the solid state, for example, by mixing MoS_2_ powder with lithium borohydrate^37^ or by vapor phase methods, where the synthesis of lithiated MoS_2_ is achieved by the diffusion of vaporized precursors.^33,38^

Here, we present a new approach for the in-growth intercalation of the MoS_2_ films grown on *c*-plane sapphire substrates. Lithium was incorporated into the films by solid-state diffusion during the sulfurization process using lithium sulfide (Li_2_S) as a source of Li. We used two different growth routes, either two-step or three-step one-zone sulfurization of Li-MoS_2_. In the two-step growth process, Mo is first deposited on the sapphire substrate and then sulfurized in the CVD chamber by annealing in a mixture of sulfur and lithium sulfide powders. In the three-step process, the Mo precursor layer is first converted into MoS_2_ and then annealed in the CVD chamber with a mixture of the two powders. Three different layer thicknesses (4, 12, and 40 nm) and two different amounts of Li_2_S were used in order to investigate the correlation between the Li source quantity and the amount of Li incorporated. In addition, we investigated the influence of lithium on the structural properties of the as-prepared Li-doped MoS_2_ films. The application of our lithiation method was found to lead to the formation of epitaxially aligned MoS_2_ films. Moreover, a conversion from vertical to horizontal alignment was observed after increasing Li doping in 12 nm thick MoS_2_ films.

## Experimental Section

### MoS_2_ and Li-Doped MoS_2_ Film Fabrication

MoS_2_ thin films were prepared using a two-step method. First, DC magnetron sputtering in an Ar atmosphere (10^–3^ mbar) from a Mo target at room temperature was employed to fabricate Mo layers (1, 3 and 10 nm thick). The DC power and emission current were set to 140 W and 0.3 A, respectively. The thickness of the as-prepared Mo films was controlled by adjusting the rotation speed of the sample holder and subsequently checked by X-ray reflectivity measurements. Some of the Mo layers were left as-prepared, while some of the pre-deposited Mo layers were sulfurized in a custom-designed CVD chamber with a substrate and sulfur powder (0.5 g) placed at the same position and temperature in the center of the furnace – so-called one-zone sulfurization.^27^ The annealing temperature, time, and heating ramp were 800 °C, 30 min, and 25 °C/min, respectively. The thickness of the MoS_2_ films after sulfurization was found to be four times larger than that of the of initial Mo layer.^26^

The process for lithium doping was carried out as follows: initial films, including Mo (1, 3 and 10 nm thick) used in a two-step method and MoS_2_ (4, 12 and 40 nm thick synthesized from 1, 3, and 10 nm thick Mo) used in a three-step method, were annealed in a mixture of sulfur and lithium sulfide. Two different amounts of Li_2_S (0.1 and 0.25 g) were used, replacing 20 and 50% of sulfur powder. The annealing parameters were the same as those used to fabricate MoS_2_ films.

### Structural Analyses

A structural analysis of the MoS_2_ layers was performed by X-ray diffraction (XRD) in a symmetrical θ/2θ configuration by a diffractometer Bruker D8 DISCOVER equipped with a rotating anode (Cu-Kα) and operating at a power of 12 kW. The crystallographic orientation and the texture of the films were studied by the azimuthal (φ-scan) measurements.

The crystallographic unit cell orientation of the MoS_2_ samples was evaluated using a grazing-incidence wide-angle X-ray scattering (GIWAXS). A home-built system based on a micro-focus X-ray source (CuKα, IμS, Incoatec), and a two-dimensional X-ray detector (Pilatus 100 K, Dectris) was used to collect the GIWAXS patterns. The angle of incidence on the sample was set to 0.2°. A sample-detector distance 90 mm was established, and it was validated by a calibration standard (corundum).

### Chemical Composition Analyses

#### Raman Spectroscopy

Raman measurements were performed using a confocal Raman microscope (Alpha 300R, WiTec, Germany) using a 532 nm excitation laser. The laser power was kept as low as 1 mW to avoid laser-induced damage. The scattered Raman signal was collected by a 50× (NA = 0.8) microscope objective and detected by a Peltier-cooled EMCCD camera. For dispersing the Raman spectra, a blazed grating with 1800 grooves/mm was employed. The energy resolution of the entire Raman spectra is approximately 0.75 cm^–1^. The Raman spectra were acquired under ambient conditions.

#### Elastic Recoil Detection Analysis (ERDA)

ERDA was used to analyze the sample composition. For the first batch of samples, a 43 MeV Cl^7+^ beam was used, and the recoil atoms and scattered ions were detected at a scattering angle of 29.5° with a Bragg Ionization Chamber, which enables energy measurement and Z identification of the particles. H and Li recoils were detected with a separate solid-state detector at a scattering angle of 40°. This detector was equipped with a 25 μm Kapton foil to stop scattered ions and heavy recoil ions. The measurements were analyzed with the software package NDF V9.3g.^39^ For the other samples, a 15 MeV Cl^4+^ beam was used, and the recoils and ions were detected at a scattering angle of 40° with a time-of-flight – energy (ToF-E) telescope, which enables energy measurement and mass identification of the particles. The detector efficiency for Li was calibrated with a separate measurement because it is less than one but with a depth resolution superior to other systems. The measurements were analyzed with the software package Potku.^40^

#### Soft X-ray Photoelectron (XPS) and X-ray Absorption Near-Edge Structure (XANES) Spectroscopy

The measurements were carried out at the BACH beamline of CNR at the Elettra synchrotron facility (Trieste, Italy).^41,42^ The XPS spectra were obtained at the photon energies of 120, 270, and 600 eV, using a Scienta R3000 hemispherical analyzer placed at an angle of 60° with respect to X-ray incidence direction. The total instrumental energy resolution was below 0.2 eV. The incoming X-rays were linearly polarized with the polarization vector lying in the scattering plane. The XPS data were collected in a normal emission geometry at a take-off angle of 90°. To minimize the effect of surface charging during data acquisition, the electric conductivity of the samples was increased by keeping them at a temperature between 150 and 200 °C. Binding energies were referenced to the S 2p_3/2_ peak of thin MoS_2_ films (161.95 ± 0.05 eV).^31,38,43,44^ The areas of the XPS peaks were corrected for photon flux and photoionization cross section.^45,46^

Li K-edge XANES spectra were acquired in total electron yield (TEY) mode by measuring the drain current through the sample using a picoammeter. The energy resolution was better than 50 meV, and the intensities were normalized to the photon flux derived from the total photoelectric current recorded at the last mirror of the beamline. The photon energy scale was calibrated by measuring Fermi level photoemission from a gold foil. The obtained XANES data were processed using the Athena and Arthemis software packages.^47^ For the XANES calculations, the ab initio real-space full multiple-scattering code FEFF9^48^ was used. FEFF is a fully relativistic, all-electron Green function code that utilizes a Barth-Hedin formulation for the exchange–correlation part of the potential and Hedin–Lundqvist self-energy correction. In our FEFF calculations, all atoms within an atomic distance of 7.5 Å from the photon absorbing Li atom were considered for the modeled crystal structures. The different structures used as input for the FEFF9 calculations were relaxed using density-functional theory and the plane-wave VASP code;^49^ all models consisted of slightly less than 100 ions. For all structures, projector-augmented wave pseudopotentials were used with a plane wave energy cut-off of 520 eV.^50^ Integration in k-space was carried out using a 2 × 2 × 2 Gamma centered Monkhorst-Pack grid.

#### Optical Measurements

Reflectance and transmittance spectra were measured with a Shimadzu SolidSpec-3700 spectrophotometer in the UV–VIS range.

## Results

### Undoped MoS_2_ Layers

**Figure 1:**
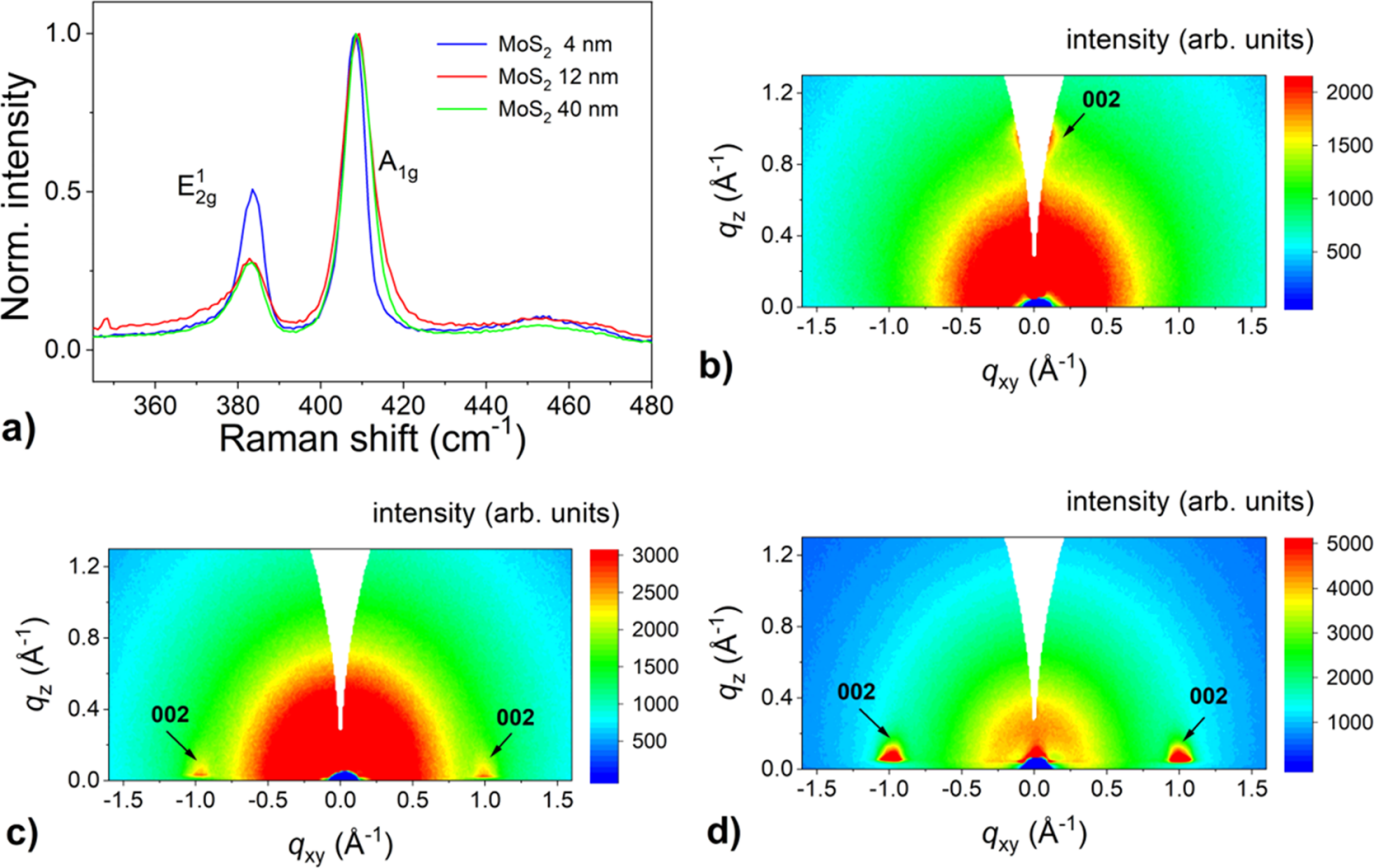
(a) Normalized Raman spectra of undoped MoS_2_ films with different thicknesses grown by one-zone sulfurization at 800 °C for 30 min on the *c*-plane sapphire substrate. Corresponding GIWAXS reciprocal space maps for 4 nm (b), 12 nm, (c) and 40 nm (d) thick MoS_2_ films.

The formation of MoS_2_ was verified by Raman spectroscopy. Figure 1a shows the region of the Raman spectrum containing the lines related to MoS_2_, namely the *E*_2g_^1^ mode at approximately 383 cm^–1^ and the *A*_1g_ mode at ∼408 cm^–1^. XRD measurements identified a *c*-axis oriented 2H-MoS_2_ phase with no indication of ordering in the *a*–*b* plane. GIWAXS measurements were conducted to estimate the orientation of the basal planes. In contrast to HRTEM (high-resolution transmission electron microscopy), which is rather a local probe, XRD and GIWAXS techniques provide a statistical average over the significant portion of the sample area. Furthermore, no specific sample preparation is required. For the thinnest films (4 nm MoS_2_), one diffraction spot was observed at *q_z_* ∼ 1 Å^–1^ (Figure 1b), indicating that the films were grown horizontally with the basal planes parallel to the substrate. For thicker films (12 and 40 nm MoS_2_), two diffraction spots were observed at *q_xy_* ∼ 1 Å^–1^ indicating a vertical alignment of these films with a random in-plane orientation. This is in agreement with previously published findings that increasing the initial Mo layer thickness leads to the vertical growth of MoS_2_ films.^23,26^ In addition, an intensity decrease of the *E*_2g_^1^ peak in the normalized Raman spectrum is also consistent with the crystallographic reorientation of the film.^51^

### Two-Step Synthesis of Lithiated MoS_2_ Films

In the two-step growth process, lithiated MoS_2_ films were synthesized in the same manner as undoped MoS_2_. First, metallic Mo layers (1, 3, 10 nm thick) were deposited on the sapphire wafers. In the second step, the part of the sulfur powder used for sulfurization annealing process was replaced by Li_2_S. Two concentrations of Li_2_S corresponding to 20% (0.1 g) and 50% (0.25 g) sulfur substitution were used. An image depicting the films after the lithiation process can be found in the Supplementary Material (Figure S1). Lithium sulfide is a solid compound with a melting point of about 938 °C. For this reason, we used a sulfurization temperature of 800 °C. We revealed that at this temperature and under a sulfur reduction atmosphere, Li_2_S evaporates, and no traces of the compound remain in the crucible after the reaction. We also conducted tests at lower annealing temperatures (400 and 600 °C); however, the Li_2_S evaporation rate was significantly reduced. Therefore, we decided to use a temperature of 800 °C for the sulfurization with Li_2_S.

**Figure 2:**
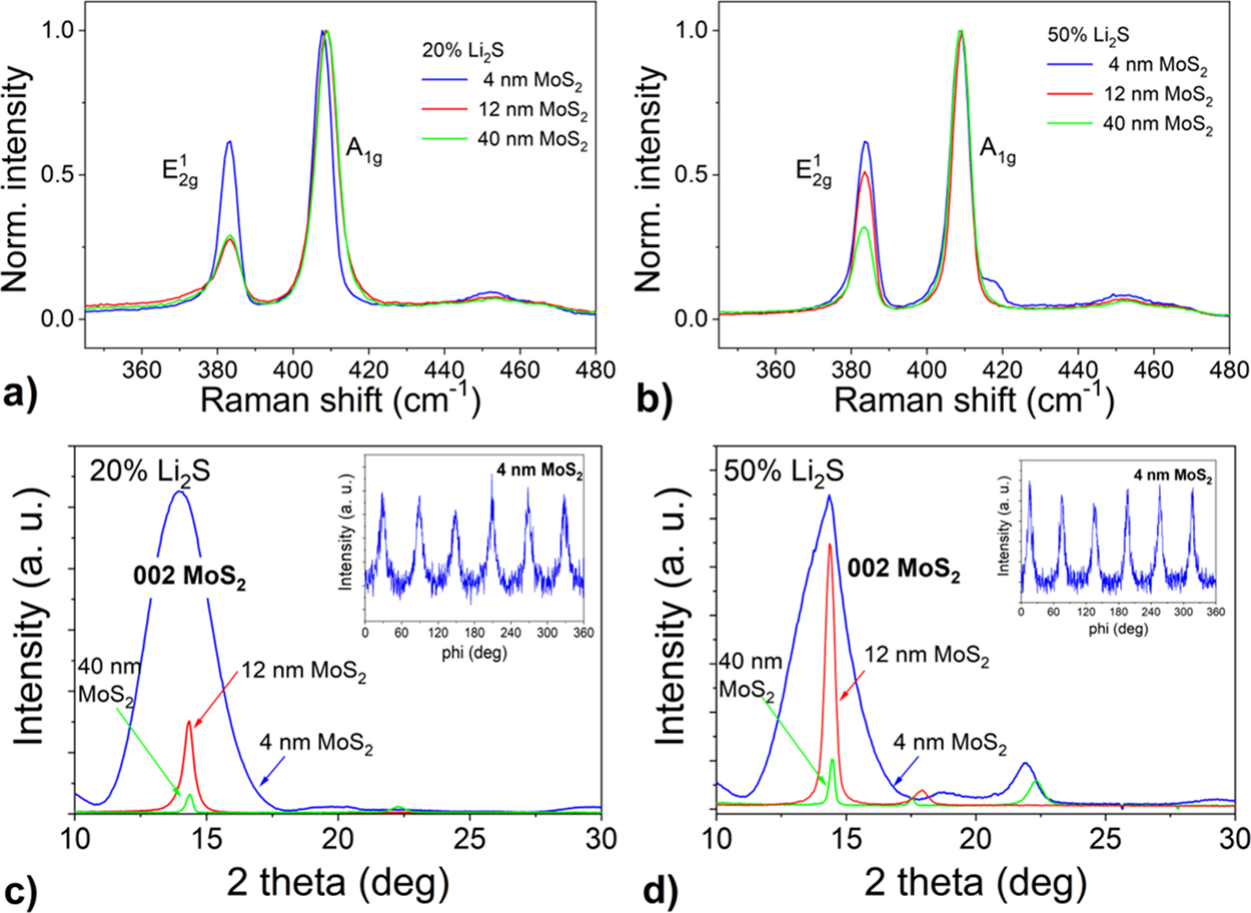
Normalized Raman spectra of lithiated MoS_2_ films with different thicknesses grown in two steps by one-zone sulfurization at 800 °C for 30 min on the *c*-plane sapphire substrate with 20% (a) and 50% (b) Li_2_S portion. XRD patterns of the same films with 20% (c) and 50% (d) Li_2_S portion. Azimuthal φ-scans of 103 diffraction of 4 nm lithiated MoS_2_ are shown as insets.

The conversion of Mo to MoS_2_ was confirmed by Raman spectroscopy. Figure 2a,b shows the normalized Raman spectra of the as-prepared films. In all cases, MoS_2_ was formed, as confirmed by the presence of characteristic *E*_2g_^1^ and *A*_1g_ peaks. The varying intensities of the *E*_2g_^1^ intensities in the normalized spectra suggest different orientations of the MoS_2_ layers, especially for the 12 nm thick films and with different nominal Li_2_S powder concentrations. The crystal structure was examined by XRD measurements in a symmetrical configuration (Figure 2c,d). For 4 nm films, a very wide 002 peak of 2H-MoS_2_, with a full width at half-maximum (FWHM) of more than 2°, dominated the pattern. The peak broadening is caused by low film thickness. The presence of a single MoS_2_ peak in the pattern is consistent with the dominant horizontal orientation of the as-prepared films. In contrast, for the case of the thickest films (40 nm), the 002 peak has a very low intensity in the symmetrical scan. We speculate that this can be a consequence of the predominant vertical alignment in the films. Focusing on the 12 nm MoS_2_ film, the peak intensity of the film prepared with a larger amount of Li is much higher than that prepared with 20% Li_2_S, due to different MoS_2_ orientation.

The in-plane ordering of the lithiated MoS_2_ layers was determined from φ-scans. We selected the strongest 103 diffraction of the hexagonal MoS_2_ phase for the analysis. We observed an epitaxial ordering only in the case of the thinnest films using both Li concentrations (see insets in Figure 2c,d). Six peaks separated by 60° observed in the pattern correspond to the diffractions coming from 6 equivalent planes: (103), (013), (1̅13), (1̅03), (01̅3), and (11̅3) and confirm the hexagonal symmetry of MoS_2_. The presence of distinct maxima in φ-scans indicates the tendency of the layers to grow epitaxially. This tendency has not been previously observed for undoped films. We hypothesize that the presence of lithium during the sulfurization process promotes the epitaxial growth of horizontally aligned MoS_2_ films.

**Figure 3:**
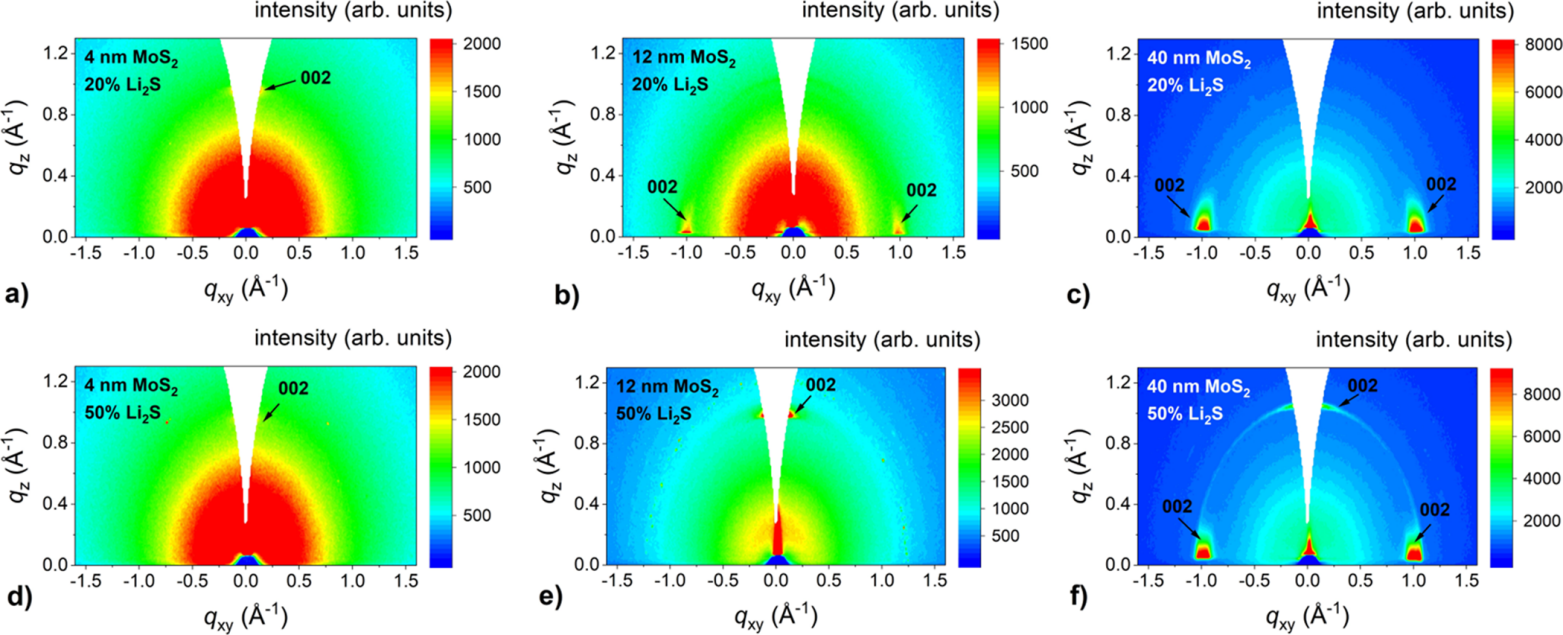
GIWAXS reciprocal space maps of lithiated MoS_2_ films with different thicknesses (4, 12, and 40 nm) grown in two steps by one-zone sulfurization at 800 °C for 30 min on the *c*-plane sapphire substrate with 20% (a, b, and c) and 50% (d, e, and f) Li_2_S portion.

The orientation of lithiated MoS_2_ films was examined by GIWAXS measurements (Figure 3). As expected, the horizontal alignment was observed for the films prepared from 1 nm thick Mo. For the case of the 3 nm thick initial layer, vertically aligned films grew when a smaller amount of Li_2_S was used. However, horizontally oriented lithiated MoS_2_ layers were obtained using a 50% of Li_2_S powder composition. For the thickest initial Mo layers, vertical alignment was present for the 20% Li_2_S concentration and mixed vertical and horizontal alignment when a 50% Li_2_S concentration was used. Thus, the presence of lithium resulted not only in the tendency of the films to grow epitaxially, but it also favored horizontal alignment of the lithiated MoS_2_ films.

Although the MoS_2_ films were prepared from sputtered Mo layers, Mo can easily oxidize in air. Therefore, the initial layer is composed of both Mo and MoO_*x*_.^52,53^ In some cases, the sulfurization of molybdenum oxide results in the horizontally aligned phase rather than the vertical one. Hutár et al.^54^ studied the influence of the oxidation level of the initial Mo layer on the orientation of the final MoS_2_ films. In this case, vertically aligned MoS_2_ was formed from the Mo layer stored in the air. Horizontal MoS_2_ growth occurred only when the Mo layer was subjected to targeted ozone exposure. To eliminate the potential influence of natural oxidation, we ensured that the pre-deposited Mo layers were exposed to air for an equal duration for both MoS_2_ and Li-MoS_2_.

### Three-Step Synthesis of Lithiated MoS_2_ Films

**Figure 4:**
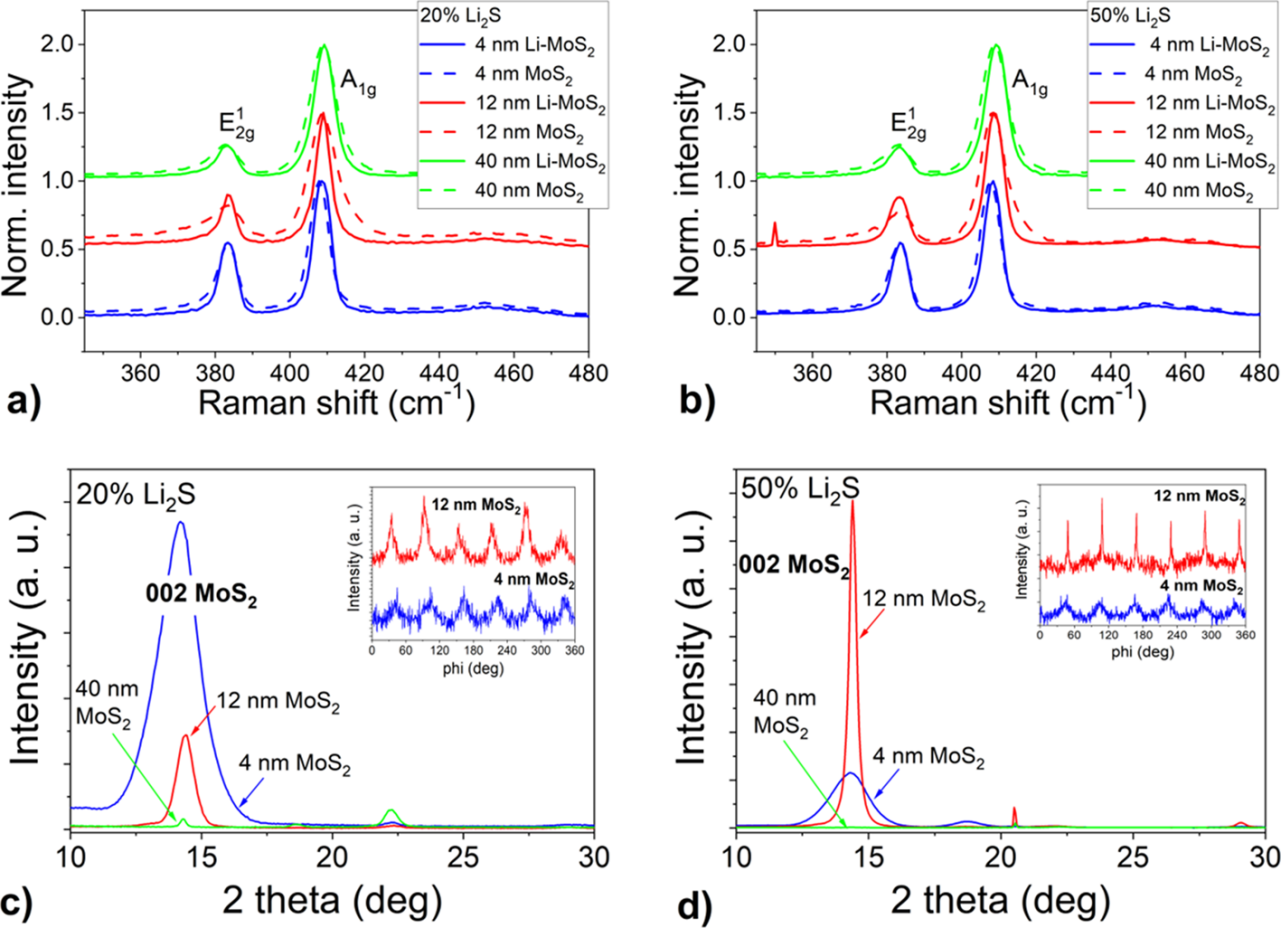
Normalized Raman spectra of lithiated MoS_2_ films with different thicknesses grown in three steps by one-zone sulfurization at 800 °C for 30 min on the *c*-plane sapphire substrate from the MoS_2_ initial layer with 20% (a) and 50% (b) Li_2_S portion. XRD patterns of the same films with 20% (c) and 50% (d) Li_2_S portion. Azimuthal φ-scans of 103 diffraction of 4 and 12 nm lithiated MoS_2_ are shown as insets.

The second MoS_2_ lithiation method involved using undoped MoS_2_ films as an initial layer for Li doping. These films were annealed in a mixture of sulfur and lithium sulfide powder (20 and 50% Li_2_S) under the same conditions as the Mo initial layers. Normalized Raman spectra of the MoS_2_ films before and after Li doping are shown in Figure 4a,b, respectively. Minimal changes were observed in all cases, except for the 12 nm thick MoS_2_ film, where the *E*_2g_^1^ peak intensity increased compared to the intensity of the undoped film. As shown below, the crystallographic alignment of this MoS_2_ film transformed from vertical to horizontal due to Li doping.

XRD measurements (Figure 4c,d) show similar results to those observed in the case of lithiated films prepared from a molybdenum initial layer. However, a very intense 002 MoS_2_ peak was observed for the samples prepared from 12 nm thick MoS_2_, especially when 50% Li_2_S was used. This suggests a horizontal orientation of lithiated films, thus indicating a conversion from vertical to horizontal alignment. Moreover, epitaxial ordering was observed not only for the thinnest films but also for 12 nm Li-MoS_2_ (see the inset in Figure 4c,d).

**Figure 5:**
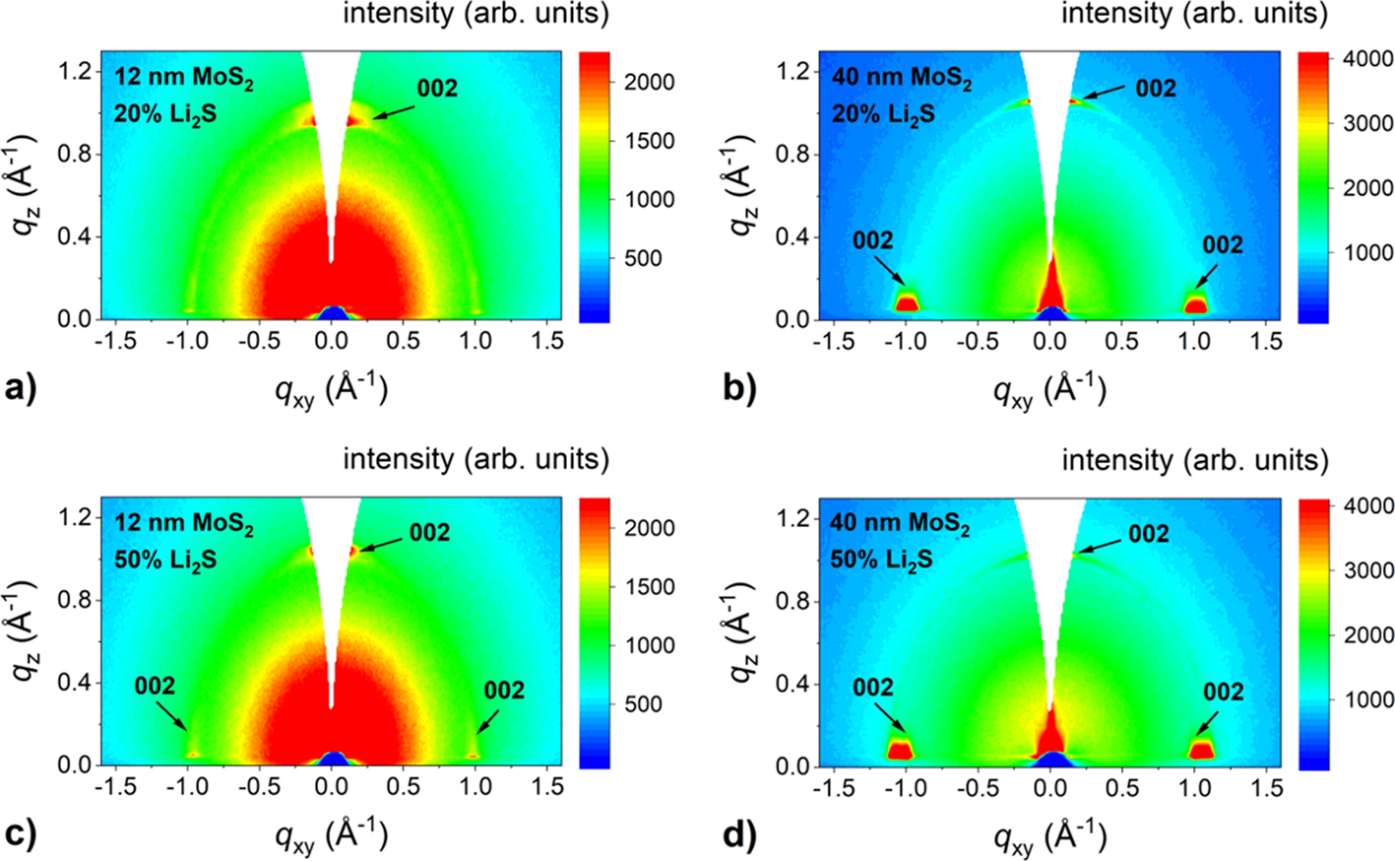
GIWAXS reciprocal space maps of lithiated MoS_2_ films with different thicknesses (12 and 40 nm) grown in three steps by one-zone sulfurization at 800 °C for 30 min on the *c*-plane sapphire substrate with 20% (a, b) and 50% (c, d) Li_2_S portion.

To investigate the film orientation and to confirm the conversion of the vertical to horizontal phase, GIWAXS measurements were carried out (Figure 5). These measurements confirmed that the orientation of the 12 nm MoS_2_ films changed from vertical (Figure 1c) to predominantly horizontal after sulfurization with both Li_2_S concentrations (Figure 5a,c). In the thickest films (Figure 5b,d), a mixture of both phases was observed, with the vertically aligned phase dominant. The change in the orientation of the already-formed 12 nm MoS_2_ film after lithiation is surprising.

To check whether the change in orientation was influenced by the second annealing step or the presence of lithium, we annealed the samples in a pure sulfur atmosphere without the presence of lithium. We did not observe any change in the film orientation (see Supplementary Material, Figure S2). This confirms the role of lithium in the reorientation of the MoS_2_ few-layer films. We had expected lithium to diffuse more easily between atomic layers in the vertically aligned films. Instead, our results suggest that above a certain thickness, the vertically aligned phase is unstable under lithium atmosphere and a film reorganization occurs. For thicker films (40 nm), only a part of the vertical phase is converted to the horizontal phase. We suspect that lithium acts as a catalyst and enables a structural reorganization. This scenario is supported by the fact that we did not observe any change in the film orientation when the lithiation took place at 600 °C (see Supplementary Material, Figure S3). We hypothesize that a certain minimum temperature and Li content are necessary for the layer conversion to occur. Nevertheless, further investigation is required to gain a better understanding of the underlying mechanism.

### Chemical Composition Analyses

**Figure 6:**
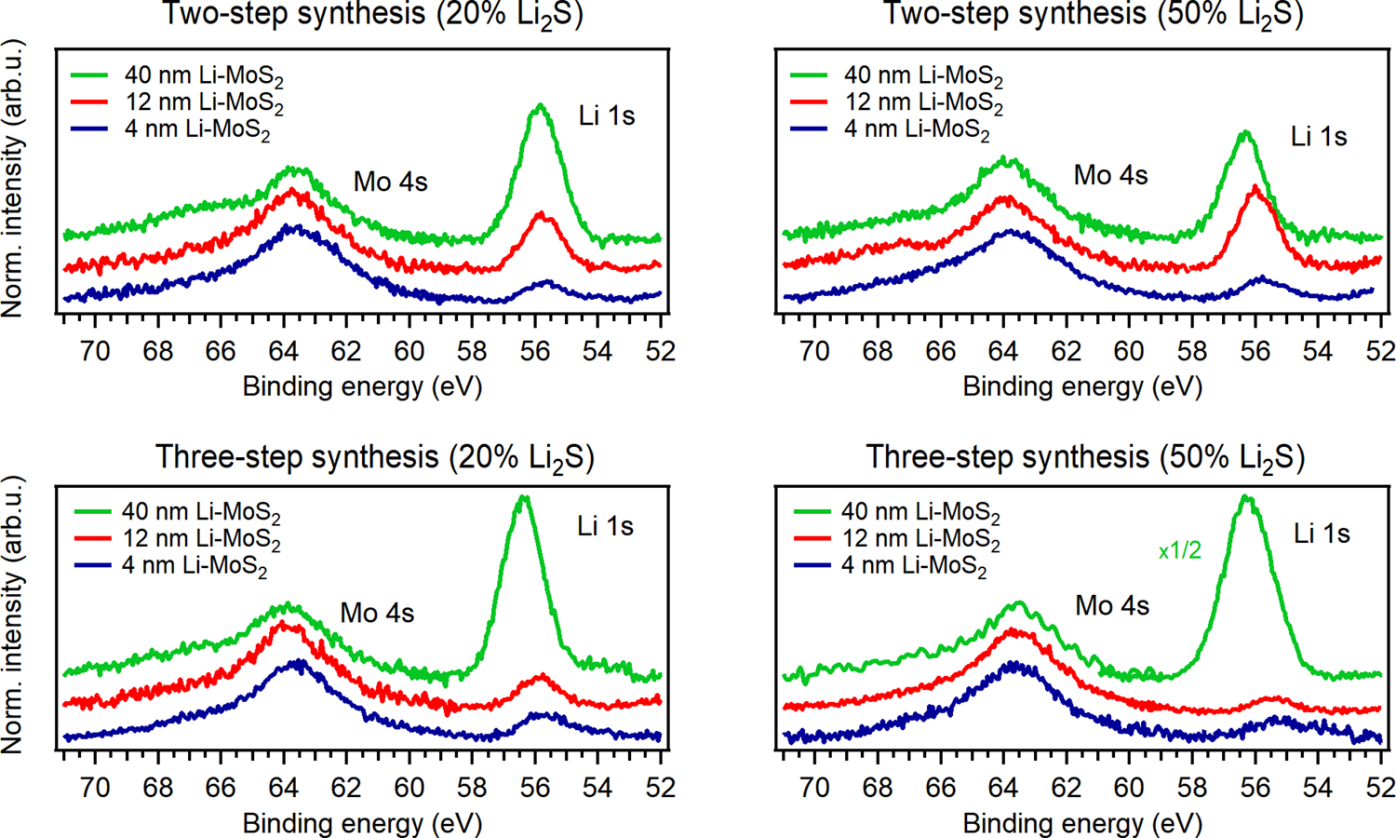
Li 1s and Mo 4s XPS spectra collected from the surfaces of Li-MoS_2_ films that were synthesized in two (top) and three (bottom) steps by sulfurization on a *c*-plane sapphire substrate with a Li_2_S portion of 20% (left) and 50% (right) Li_2_S portion. All spectra were recorded using a photon energy of 270 eV.

Confirmation of the presence of lithium as well as the chemical composition of the layers was identified by a combination of synchrotron-based soft X-ray photoemission spectroscopy (XPS), X-ray absorption near-edge structure (XANES) spectroscopy, and elastic recoil detection analysis (ERDA). Both XPS and ERDA composition analyses resulted in a S:Mo atomic ratio close to 2 and confirmed the presence of lithium for all samples. Figure 6 shows Li 1s and Mo 4s XPS core-level spectra taken from the Li-MoS_2_ samples prepared by two and three-step methods (Mo 3d and S 2p XPS spectra can be found in the Supplementary Material, Figures S4 and S5). The Li 1s peaks for the 4 and 12 nm Li-MoS_2_ samples were centered at 55.8 ± 0.1 eV, while the Li 1s peak of 40 nm Li-MoS_2_ is shifted to 56.4 ± 0.2 eV. The binding energy of 55.8 eV can be attributed to Li-intercalated MoS_2_.^44^ However, the literature shows a significant variation in the reported binding energy values for a specific phases, and several different phases exhibit nearly identical binding energies.^55^ Thus, the chemical phase assignment based on the Li 1s binding energy should be done with caution. However, our assignment of the Li 1s peak to lithiated MoS_2_ is supported by Li K-edge XANES spectroscopy, as discussed in the following. On the other hand, the observed shift of the Li 1s peak toward higher binding energies for the 40 nm MoS_2_ film suggests the presence of a different chemical phase. Through the utilization of XPS depth profiling, wherein XPS signals were collected by varying the excitation energy (refer to Supplementary Material, Table S1), we found that the shifted Li 1s component is limited to the top surface of MoS_2_, indicating the formation of a thin layer of a different Li-based compound. The presence of −SO_4_^2–^ anions, which appeared in the S 2p spectra (Figure S4) of 40 nm thick MoS_2_ films, indicates that the main compound on the surface is likely Li_2_SO_4_.

**Figure 7:**
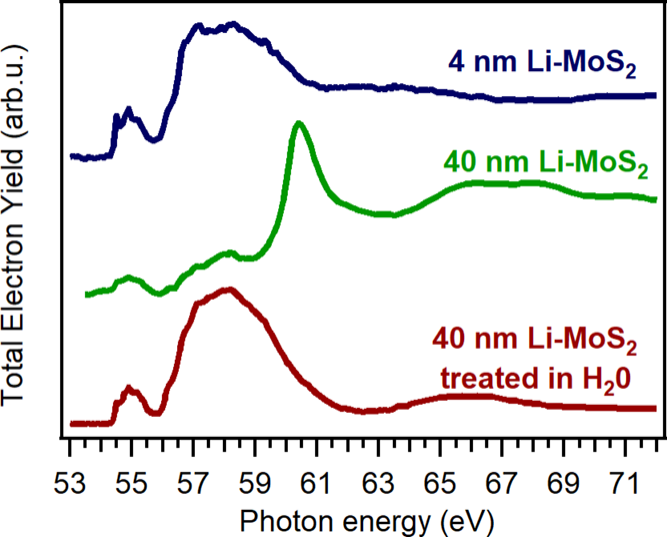
Li K-edge XANES spectra collected on the Li-MoS_2_ films prepared by two-step synthesis. The bottom spectrum was acquired on the 40 nm Li-MoS_2_ sample after it was dipped into ultra-pure water for a few minutes.

It has been demonstrated that Li K-edge XANES spectra are distinct for various lithium compounds, with specific energy positions and line shapes, that serve as unique fingerprints for identifying phase composition.^56−59^Figure 7 shows the Li K-edge spectra collected on the 4 and 40 nm thick Li-MoS_2_ films grown starting from 1 and 10 nm Mo layers. The thinner film exhibits a Li K-edge spectrum which has not been reported in the literature previously. Phases such as Li_2_S, LiOH, Li_2_CO_3_, and Li oxides can be excluded as they would exhibit certain distinct features that are not present in the observed spectrum. Thus, we ascribe the Li K-edge shape obtained from 4 nm thick Li-MoS_2_ film to the lithiated MoS_2_. The Li K-edge spectrum recorded for the 40 nm thick film is quite different, although weak features of Li-MoS_2_ in the energy range from 55 to 58 eV are still visible. The dominant features between 60 and 70 eV are attributed to Li_2_SO_4_^29^ being the main phase on the surface. However, after removing the topmost surface layers, 40 nm Li-MoS_2_ exhibited a Li K-edge spectrum that is nearly identical to the one obtained for the thinner film. This suggests that the thicker MoS_2_ films were lithiated in a similar manner to the thin ones.

**Figure 8:**
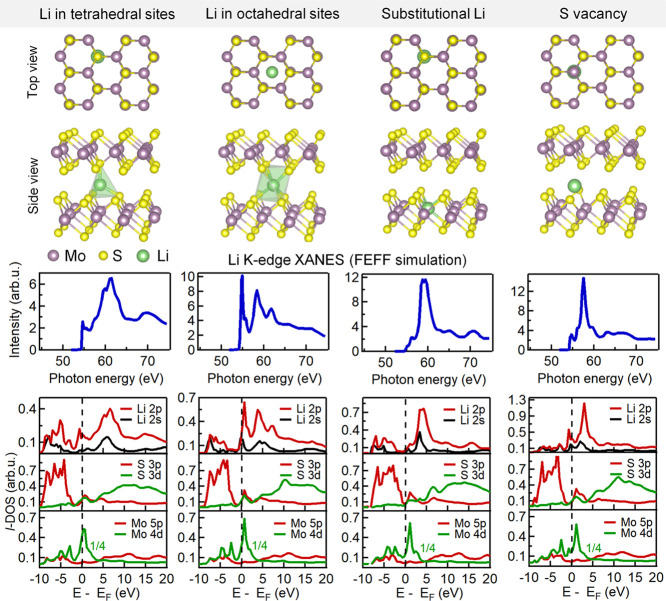
Li K-edge XANES spectra for Li doped 2H-MoS_2_ obtained by FEFF calculations. Top: fragments of the structural models for hexagonal 2H-MoS_2_ with atoms in tetrahedral and octahedral interstitial sites, substitutionally doped MoS_2_, and with lithium near a single sulfur vacancy. Middle: simulated Li K-edge XANES spectra. Bottom: calculated orbital-projected density of states for Li, S, and Mo atoms.

To support our assignment of the measured Li K-edge XANES spectra to Li-MoS_2_, we have explored the electronic structure of the unoccupied states of Li-doped MoS_2_ theoretically (Figure 8). Since none of the experimental techniques we used showed any evidence of the 1T′-MoS_2_ allotrope, only the hexagonal 2H-MoS_2_ host lattice is considered in our simulations. The layered structure of MoS_2_, in which the S–Mo–S triple layers interact by weak van der Waals bonds, allows easy intercalation of Li atoms. The intercalation sites between the sulfur atoms in the van der Waals gap can have either octahedral or tetrahedral coordination. The corresponding simulated Li K-edge spectra are presented in Figure 8. The calculated spectra for Li atoms in both interstitial sites reproduce the main experimental features, the primary edge above 57 eV as well as the pre-edge region around 55 eV. A slightly better agreement with the measured spectrum can be seen in the spectrum calculated for Li atoms in the tetrahedral sites. The Li K-edge shape is dominated by the dipole allowed excitation of the Li 1s core level electrons to Li 2p unoccupied states. The calculated orbital-projected densities of states (lDOS), plotted in Figure 8, show that the pre-edge features in the Li K-edge spectrum originate from the unoccupied Li 2p state hybridized with mixed Mo-S states near the bottom of the conduction band, whereas the region between 57 and 63 eV is dominated by unoccupied Li 2p/2s states which are partially mixed with S 3p.

We have also performed calculations for hypothetical substitutionally doped 2H-MoS_2_ with Li on Mo sites and with Li near a single sulfur vacancy. The simulated spectrum for the model with sulfur vacancy showed good agreement with the experimental spectrum. Note that the Li atom migrated rather deep inside the van der Waals gap after the structure relaxation. Similar to the intercalation Li-MoS_2_ compound, the primary edge reflects the unoccupied Li 2p state, whereas the pre-edge region corresponds to Li 2p strongly hybridized with S 3p–Mo 4d states. In summary, the theoretical calculations confirmed that the distinct Li K-edge spectral shape observed on Li-doped MoS_2_ prepared in this study corresponds mainly to Li_*x*_MoS_2_ intercalates.

Next, we examine the amount of Li incorporated in MoS_2_. The Li molar fraction can be estimated by XPS from the Mo 4s and Li 1s intensity ratio. However, in order to keep the photoemission sensitivity to Li 1s sufficiently high, photon energies below 600 eV were used. The probing depth for such low energies is less than 4 nm for MoS_2_. Therefore, the Li concentration was also examined by ERDA, which provided us with information on the elemental composition from the whole volume of the thin films prepared here. Table 1 shows the Li:Mo ratios calculated from ERDA measurements compared with the results obtained from XPS.

**Table 1:** Li Atomic Concentrations

layer thickness	Li_2_S amount (%)	two-step synthesis		three-step synthesis	
		*x* (Li_*x*_MoS_2_)		*x* (Li_*x*_MoS_2_)	
		ERDA	XPS	ERDA	XPS
4 nm MoS_2_	20	0.15	0.11 ± 0.04	0.14	0.13 ± 0.02
12 nm MoS_2_	20	0.04	0.3 ± 0.1	0.63	0.17 ± 0.05
40 nm MoS_2_	20	0.05	n/a^a^	0.06	n/a^a^
4 nm MoS_2_	50	0.27	0.10 ± 0.03	2.20	0.10 ± 0.01
12 nm MoS_2_	50	0.08	0.41 ± 0.06	0.44	0.10 ± 0.05
40 nm MoS_2_	50	0.02	n/a^a^	0.02	n/a^a^

a Uneven depth distribution of Li near the surface (see Supplementary Material for more details).

For the thinnest films (4 nm Li-MoS_2_) prepared with 20% Li_2_S, the lithium content determined from XPS and ERDA was very similar for both routes. On the other hand, the lithium concentrations obtained by the two techniques differ for the other samples. We ascribe these differences to the presence of an uneven Li depth distribution in the grown layers. The 4 nm thick films prepared with a higher fraction of Li_2_S contained larger amounts of lithium inside the film according to the ERDA results. This indicates that lithium is concentrated close to the substrate and thus is not detectable by XPS. Similar results were obtained for 12 nm Li-MoS_2_ prepared by the three-step route. These films had horizontal alignment with epitaxial ordering. We assume that such a structure facilitates lithium storage inside MoS_2_ films. However, we cannot exclude the possibility that a lithium-rich interface was formed between the MoS_2_ and the sapphire substrate. On the contrary, the 12 nm Li-MoS_2_ films synthesized in two-steps exhibited less lithium in the bulk and higher concentrations in the near surface regions. The reason for the reversed concentration profile could be a different Li_2_S decomposition ratio and diffusion coefficient of Li in MoS_2_ and on metallic Mo. For the thickest films (40 nm MoS_2_), we also observed that most of the Li was concentrated on the surface. All thick films had a dominantly vertical alignment. We speculate that the intercalated Li is thus more accessible to gas molecules, and Li segregated to the surface upon interaction with residual oxygen in the CVD chamber or after the air exposure. It is worth noting that while the Li 1s XPS spectra of the 40 nm Li-MoS_2_ samples taken after a long air exposure were significantly altered, those of the 4 and 12 nm Li-MoS_2_ exhibited minor or no changes (see Supplementary Material, Figure S6). This finding supports the presence of reactive lithium species on the surface of the 40 nm thick sample.

**Figure 9:**
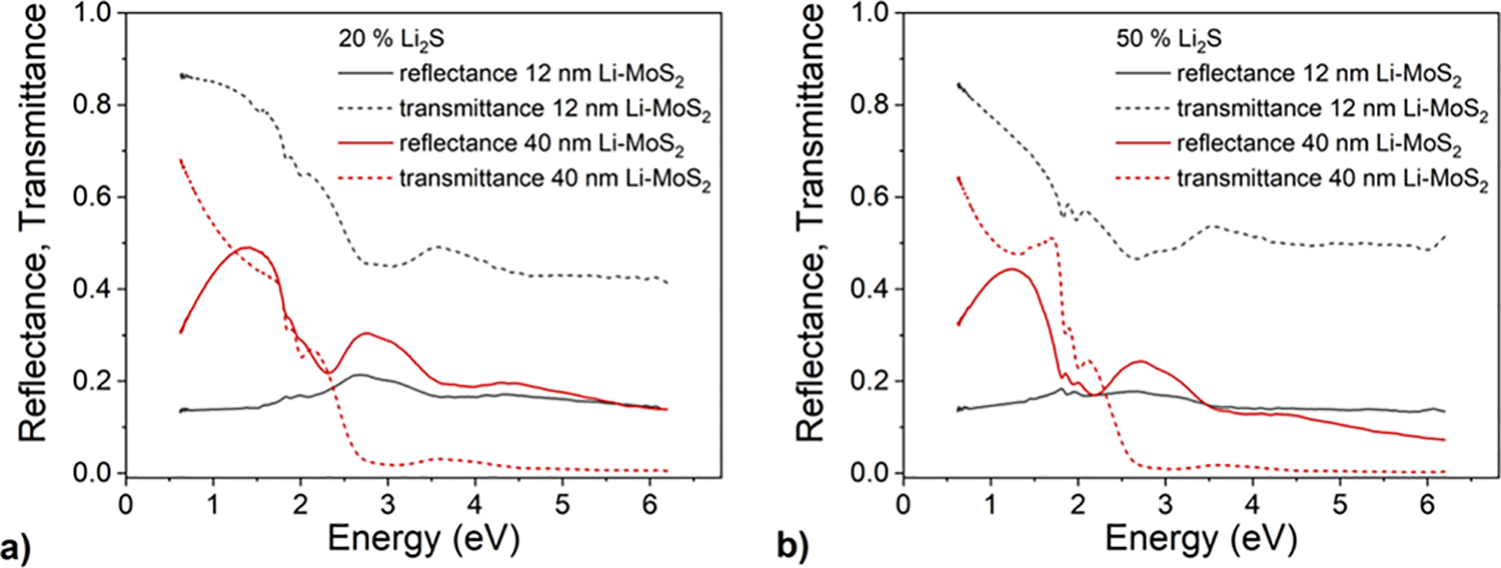
Reflectance (solid line) and transmittance (dashed line) of 12 nm (black) and 40 nm (red) thick Li-doped MoS_2_ samples prepared by a two-step method with a Li_2_S portion of 20% (a) and 50% (b).

Figure 9 shows the as-measured transmittance and reflectance spectra of 12 nm and 40 nm thick lithiated MoS_2_ layers prepared by a two-step method with 20% (Figure 9a) and 50% (Figure 9b) portion of Li on a transparent sapphire substrate. The spectra were measured from the thin film side of the sample (i.e., the light beam hits the thin layer first and then the substrate).^60^ The latter is ∼13.6% if the sapphire refractive index of 1.7 is considered. For the thinner samples, the reflectance and transmittance sum up to nearly 100% in the lowest energy part of the spectrum below ∼1.2 eV. The absorbance is negligible in that range and so is the extinction coefficient of the MoS_2_ layer. In such a case, it can be shown that the reflectance of the sample converges to the sapphire substrate reflectance in the limit *ν* → 0.^61^ If we take into account the sapphire refractive index *n*_3_ = 1.7, the latter amounts to approximately 14%.

Above ∼1.7 eV, the absorption of the MoS_2_ layer sets in, visible in the spectrum as a decrease in the sample’s transmittance. Two excitonic peaks can be seen in the reflectance and transmittance spectra as weak features at around 1.9 eV. At even higher energies, the spectra’s shape is given by the dispersion of the MoS_2_ complex refractive index, which reflects the electronic band structure of MoS_2_.

The existence of a feature below 1.7 eV that manifests itself as a broad maximum (in reflectance) and minimum (in transmittance) makes the spectra of the thicker samples significantly different from those of thinner ones. Even though the layer absorption is undoubtedly not negligible in this range, the feature is due to a thin film interference from the MoS_2_ layer. The reflectance at the maximum is given by (*n*_2_^2^ – *n*_3_)^2^/(*n*_2_^2^ + *n*_3_)^2^.^61^ Taking the values of 0.43 and 0.49 for the reflectance, we get *n*_2_ = 2.86 and 3.10 for the sample with 20% Li_2_S and 50% Li_2_S, respectively. The position of the reflectance maxima can be calculated from 1/4*n*_2_*d*, giving the values in the range of 2.5–2.7 eV, much higher than those observed. On the other hand, the maxima are asymmetric, with their high-frequency parts cut off as the layers begin to absorb significantly. This shifts the maxima′s positions to lower energy and reduces their amplitudes. Therefore, the real refractive index of the two samples is expected to have larger values than those calculated above.^62−64^ The strong layer absorption also suppresses thin-film interference maxima and minima in the visible and UV spectral ranges.

The position and the intensity of the excitonic peaks at 1.9–2.0 eV are sensitive to high levels of Li doping.^65,66^ However, the optical spectra in Figure 9 do not show any substantial changes compared to undoped MoS_2_, indicating that our samples are still in the low doping regime.

## Conclusions

Layered molybdenum disulfide has a unique ability to intercalate guest species into its van der Waals gap, which opens opportunities to tune the physical and chemical properties of thin MoS_2_ films in a controlled manner. To shift from fundamental to practical explorations, scalable synthesis approaches that allow for controlled physical dimensions and chemical composition are essential. In this work, we developed and investigated a new method to incorporate lithium into thin MoS_2_ films. We investigated structural modification, chemical changes, and optical properties in the lithiated few-layer MoS_2_. Our study demonstrates that the one-zone sulfurization approach with the addition of lithium sulfide is a suitable method for fabricating Li-doped MoS_2_ films with varying thicknesses. The presence of the lithium was confirmed by ERDA and XPS measurements, with an average concentration of lithium of about 6% depending on the film thickness, orientation, and lithiation conditions. For practical applications, it is a challenge to maintain stable Li doping of transition metal dichalcogenides. Therefore, it is worth noting that the horizontally oriented Li-doped MoS_2_ films prepared using our new approach have demonstrated chemical stability over several months, with no evidence of leakage or chemical degradation. XRD and GIWAXS measurements revealed that lithium in the reaction system influences the reaction path during the growth process by promoting epitaxial ordering in the doped films and inducing vertical-to-horizontal conversion in vertically aligned MoS_2_ upon lithiation. We hypothesize that lithium acts as a catalyst in facilitating this conversion. So far, the initial layer thickness and sulfur vapor pressure have been considered as critical parameters in determining the final orientation of MoS_2_ thin films grown by CVD. We have demonstrated that the MoS_2_ basal plane orientation in the few-layer films can also be tuned by lithium doping. Since vertically and horizontally oriented films exhibit distinct electronic, chemical, and optical properties, controlling the crystallographic orientation of MoS_2_ will play a crucial role in engineering future devices that incorporate MoS_2_ layers.

## Data Availability

The XPS and XANES data underlying this study are openly available in Zenodo at doi:10.5281/zenodo.7709466.

## Supporting Information Available

Image of the Li-MoS_2_ films, GIWAXS reciprocal space maps of MoS_2_ films fabricated by one-zone sulfurization at 800 °C for 30 min and annealed subsequently at 800 °C for 30 min solely in sulfur environment without Li_2_S addition and lithiated MoS_2_ films prepared at 600 °C, S 2p and Mo 3d XPS spectra of Li-MoS_2_ films, and atomic concentration ratios of Li and Mo determined from Li 1s and Mo 4s XPS integrated intensities (PDF)
