# Supplementary material for: Lithium-Induced Reorientation of Few-Layer MoS2 Films
Source: Chem Mater. 2023 Aug 2;35(16):6246–57. doi: 10.1021/acs.chemmater.3c00669 (PMC10448679; doi:10.1021/acs.chemmater.3c00669)
Supplement: Supplementary file 1 — cm3c00669_si_001.pdf [file cm3c00669_si_001.pdf]

Supplementary material for

Lithium-induced reorientation of few-layer MoS_2 films

Michaela Sojková,[1] Igor Píš,[2] Jana Hrdá,[1] Tatiana Vojteková,[1] Lenka Pribusová Slušná,[1] Karol Vegso,[3,4] Peter Siffalovic,[3,4] Peter Nadazdy,[1] Edmund Dobročka,[1] Miloš Krbal,[5] Paul J. Fons,[6,7] Frans Munnik,[8] Elena Magnano,[2,9] Martin Hulman[1] and Federica Bondino[2*]*

[1] Institute of Electrical Engineering, SAS, Dúbravská cesta 9, 841 04 Bratislava, Slovakia

[2] IOM-CNR, Istituto Officina dei Materiali, S.S. 14 km – 163.5, 34149 Basovizza, Trieste, Italy

[3] Institute of Physics, Slovak Academy of Sciences, Dúbravská cesta 9, 84511 Bratislava, Slovakia

[4] Centre for advanced materials application (CEMEA), Slovak Academy of Sciences, Dúbravská cesta 5807/9, 84511 Bratislava, Slovakia

[5] Center of Materials and Nanotechnologies (CEMNAT), Faculty of Chemical Technology, University of Pardubice, Legions Square 565, 530 02 Pardubice, Czech Republic

[6] Faculty of Science and Technology, Keio University, Department of Electronics and Electrical Engineering, 223-8522 3-14-1 Hiyoshi, Kohoku-ku, Yokohama, Kanagawa 223-8522, Japan

[7] Device Technology Research Institute, National Institute of Advanced Industrial Science and Technology, 1-1-1 Umezono, Tsukuba 305-8568, Ibaraki, Japan

[8] Helmholtz-Zentrum Dresden-Rossendorf, e.V. Bautzner Landstrasse 400, D-01328 Dresden, Germany

[9] Department of Physics, University of Johannesburg, PO Box 524, Auckland Park, 2006, Johannesburg, South Africa

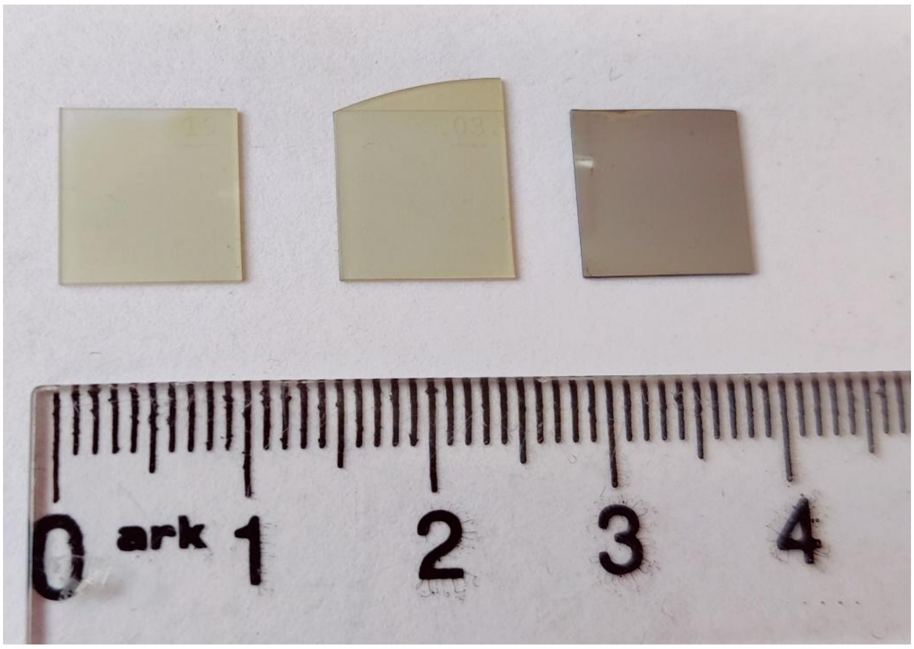

Figure S1. Image of the Li-MoS_2 films with different thickness (from left to right: 4, 12, and 40 nm) grown in two steps by one-zone sulfurization at 800 °C for 30 min on the *c*-plane sapphire substrate with 50% Li_2S portion. Small white areas at the edges are the places where the wafers were fixed to the evaporator plate.

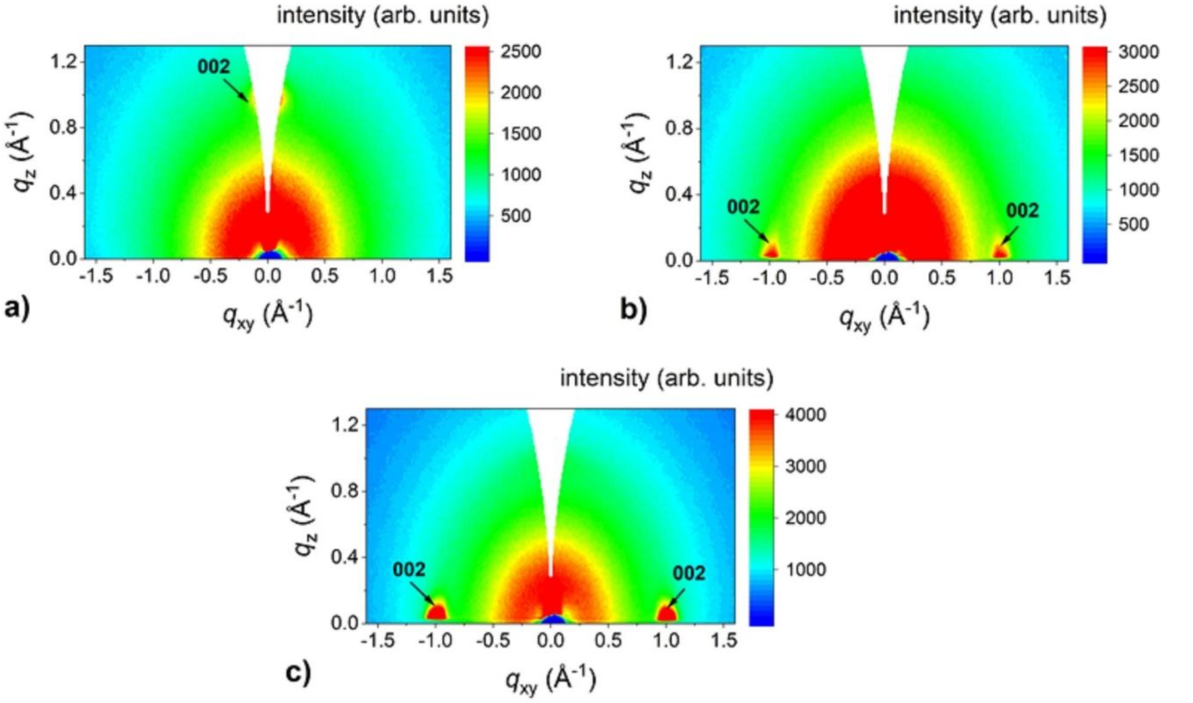

Figure S2. GIWAXS reciprocal space maps of 4 (a), 12 (b) and 40 (c) nm thick MoS_2 films fabricated by one-zone sulfurization at 800 °C for 30 min and annealed subsequently at 800 °C for 30 min solely in sulfur environment without Li_2S addition.

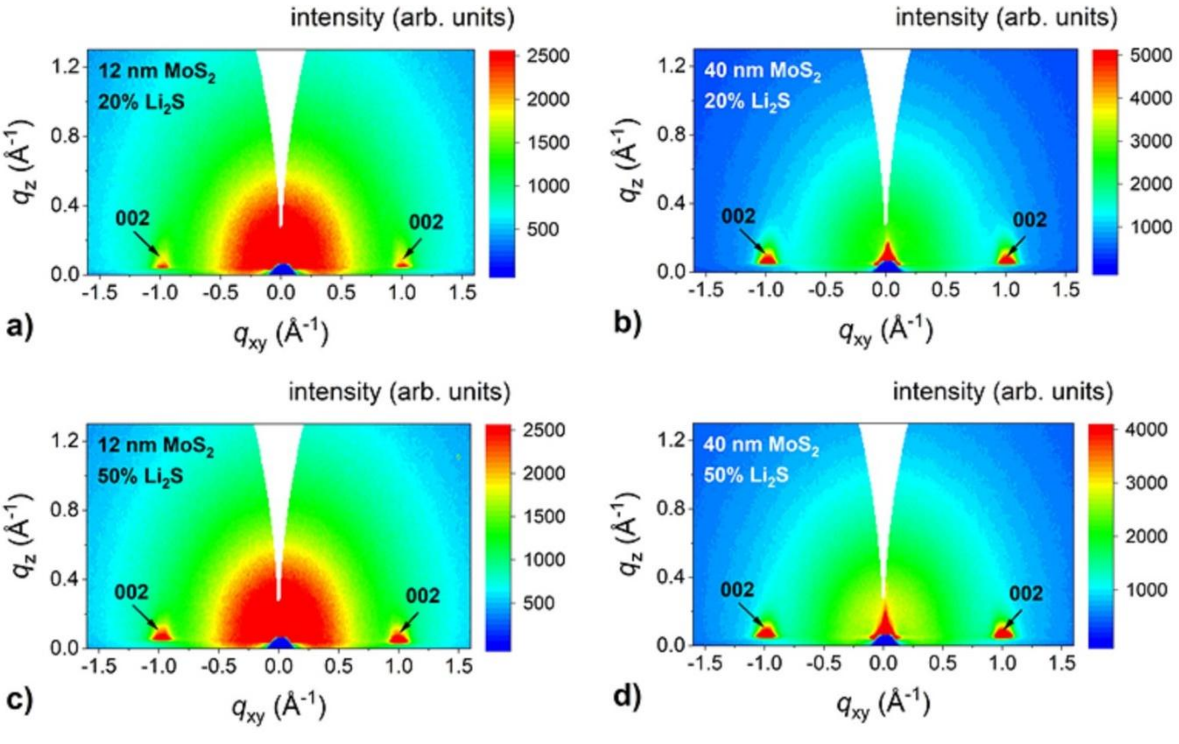

Figure S3. GIWAXS reciprocal space maps of lithiated MoS_2 films with different thicknesses (12 and 40 nm) grown in three steps by one-zone sulfurization at 600 °C for 30 min on the *c*-plane sapphire substrate with 20% (a, b) and 50% (c, d) Li_2S portion.

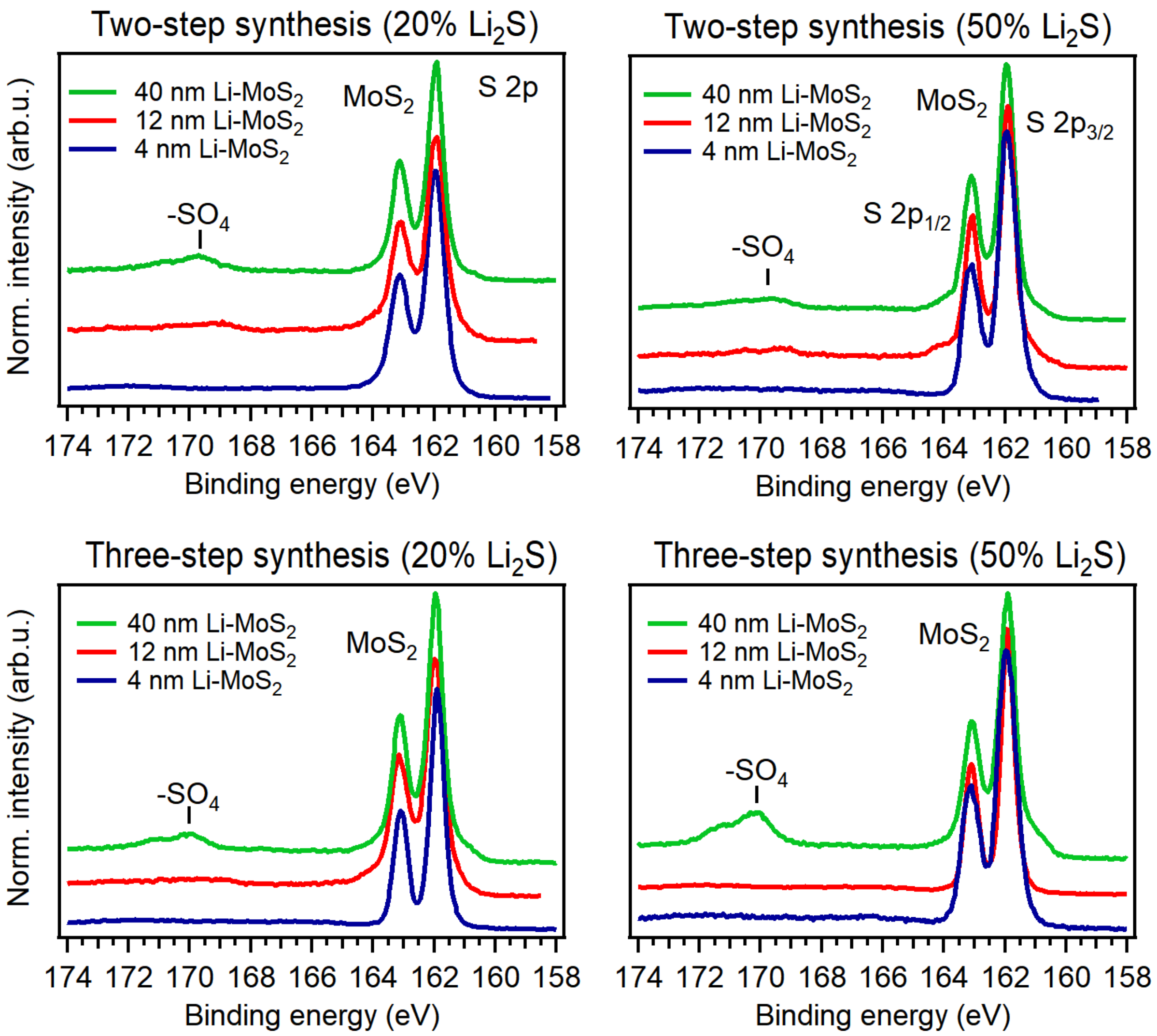

Figure S4. S 2p XPS spectra of Li-MoS_2 films synthesized in two (top) and three (bottom) steps by sulfurization on the *c*-plane sapphire substrate with a Li_2S portion of 20% (left) and 50% (right). All spectra were recorded using a photon energy of hν = 605 eV. The intensities are normalized to the main S $2p_{3/2}$ peak height.

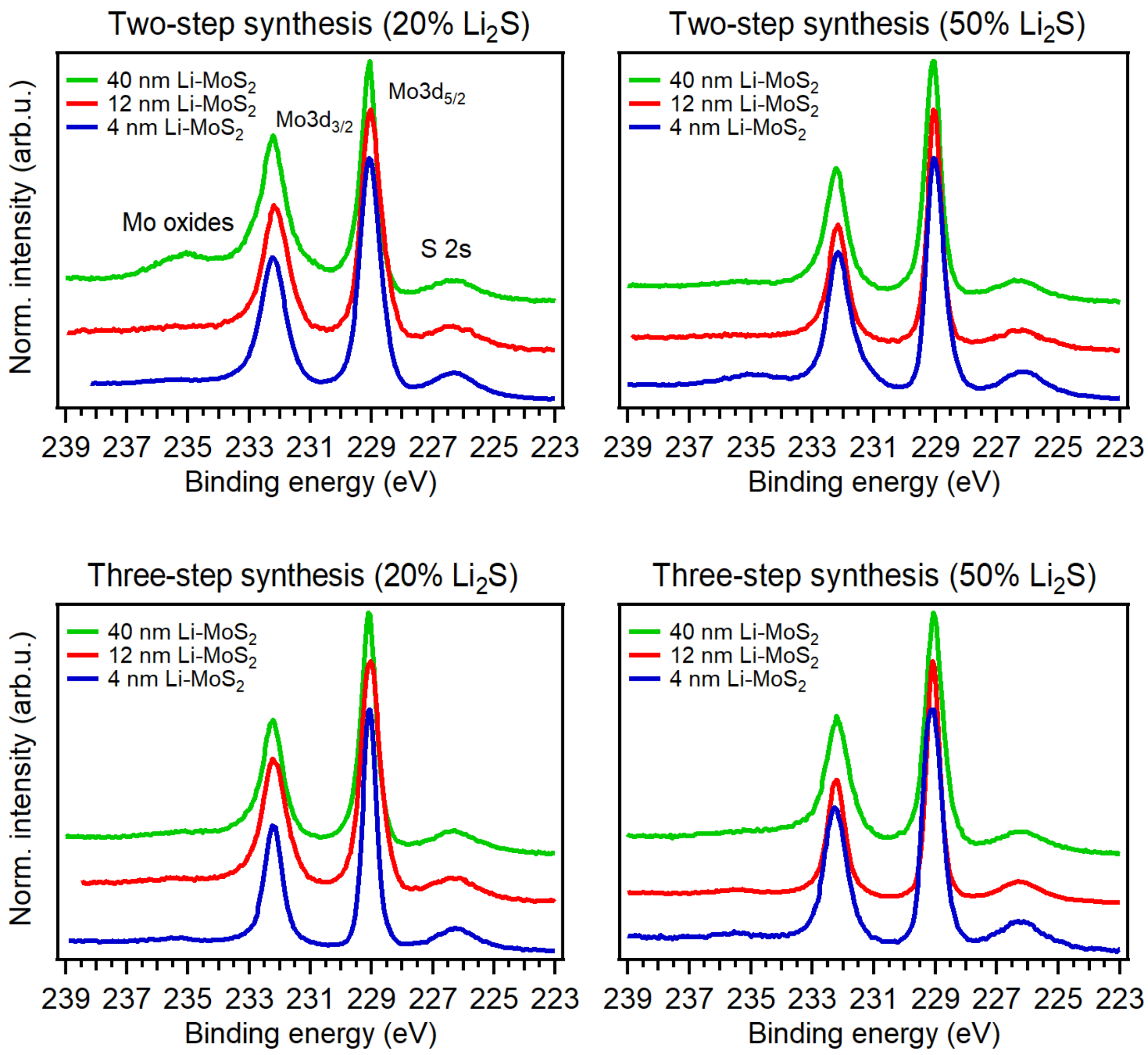

Figure S5. Mo 3d XPS spectra of Li-MoS_2 films synthesized in two (top) and three (bottom) steps by sulfurization on the *c*-plane sapphire substrate with a Li_2S portion of 20% (left) and 50% (right). All spectra were recorded using a photon energy of hν = 605 eV. The intensities are normalized to the main Mo $3d_{5/2}$ peak height.

Table S1. Atomic concentration ratios of Li and Mo determined from Li 1s and Mo 4s XPS integrated intensities recorded using photon energies corresponding to different probing depths (Λ). The probing depth is defined as the depth from which 95 % of the photoelectron signal originates (three-times the inelastic mean free path of MoS_2 calculated from the TPP-2M formula [1]). The intensities were normalized to the corresponding element sensitivity factors [2]. The average concentrations and standard deviations are listed in the last column. Note that for 40 nm thick films, the Li concentration increases with surface sensitivity. These samples were covered by thin films containing some Li_2SO_4, which hinders the determination of the amount of Li incorporated into MoS_2 by the present XPS method.

Two-step synthesis		Li:Mo concentration ratio *x* (at.%)			
**Layer thickness**	**Li_2S portion**	**hν = 605 eV** (Λ = 3.9 nm)	**hν = 270 eV** (Λ = 2.1 nm)	**hν = 120 eV** (Λ = 1.4 nm)	**Average** (Li_xMoS_2)
4 nm MoS_2	20 %	0.14	0.07	0.12	0.11 ± 0.04
12 nm MoS_2	20 %	0.33	0.22	0.42	0.3 ± 0.1
40 nm MoS_2	20 %	n/d	0.58	5.2	n/a
4 nm MoS_2	50 %	0.12	0.07	0.12	0.10 ± 0.03
12 nm MoS_2	50 %	0.44	0.34	0.46	0.41 ± 0.06
40 nm MoS_2	50 %	0.26	0.49	2.2	n/a
Three-step synthesis					
4 nm MoS_2	20 %	0.15	0.12	0.11	0.13 ± 0.02
12 nm MoS_2	20 %	0.22	0.12	0.16	0.17 ± 0.05
40 nm MoS_2	20 %	n/d	0.8	5.8	n/a
4 nm MoS_2	50 %	n/d	0.11	0.09	0.10 ± 0.01
12 nm MoS_2	50 %	0.15	0.05	0.09	0.10 ± 0.05
40 nm MoS_2	50 %	1.6	1.9	14	n/a

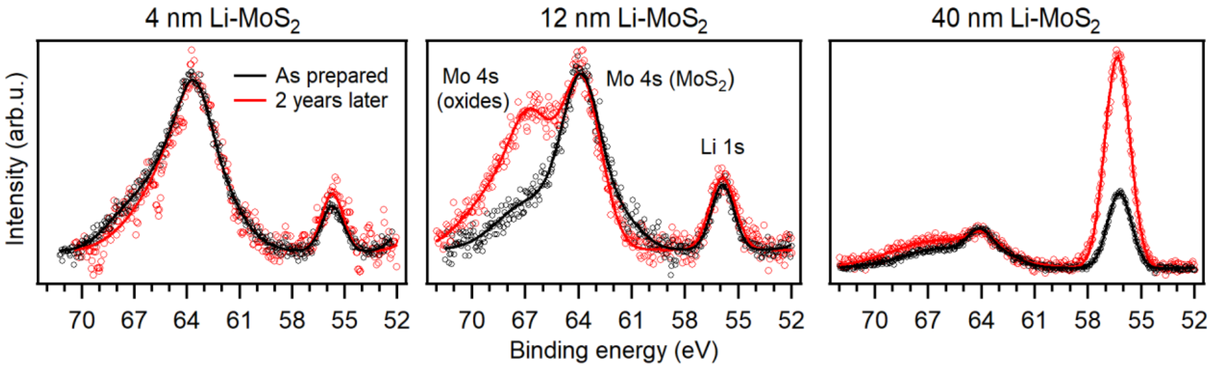

Figure S6. Li 1s and Mo 4s XPS spectra measured on Li-MoS_2 samples (three-step synthesis, 20% Li_2S portion) after a long air exposure. The thin 4nm Li-MoS_2 sample exhibited practically no change. Sample 12 nm Li-MoS_2 showed some molybdenum oxides, but the concentration ratio between Li and Mo in MoS_2 remained unchanged. The oxides are on the surface, and can be removed by ion sputtering or dipping the sample into water. The intensity of Li on the thick 40 nm Li-MoS_2 sample increased, most probably due to Li segregation from the subsurface region. The spectra were recorded using a photon energy of hν = 270 eV.

References

[1] S. Tanuma, C.J. Powell, D.R. Penn, Calculations of electron inelastic mean free paths. V. Data for 14 organic compounds over the 50-2000 eV range, Surface and Interface Analysis. 21 (1994) 165–176. doi:10.1002/sia.740210302.

[2] J.J. Yeh, I. Lindau, Atomic subshell photoionization cross sections and asymmetry parameters: 1 ≤ Z ≤ 103, Atomic Data and Nuclear Data Tables. 32 (1985) 1–155. doi:10.1016/0092-640X(85)90016-6.
